# Retraction: Associations of porphyromonas gingivalis infection and low beclin1 expression with clinicopathological parameters and survival of esophageal squamous cell carcinoma patients

**DOI:** 10.3389/pore.2023.1611617

**Published:** 2023-12-14

**Authors:** Pathology and Oncology Research Editorial Office

**Affiliations:** Frontiers Media SA, Lausanne, Switzerland

The journal retracts the 08 December 2021 article cited above.

Following publication, concerns were raised regarding data misrepresentation. In particular, multiple discrepancies were identified in the flow cytometry graphs, western blots and bar graphs in Figure 4. Following provision of raw data by the authors, the Editor in Chief concluded that the article’s conclusions and assertions were not sufficiently supported by the findings from the material provided; therefore, the article has been retracted. The authors do not agree to this retraction.

